# Structure and diversity of native bacterial communities in soils contaminated with polychlorinated biphenyls

**DOI:** 10.1186/s13568-020-01058-8

**Published:** 2020-07-10

**Authors:** Adalberto Zenteno-Rojas, Esperanza Martínez-Romero, Daniel Castañeda-Valbuena, Clara Ivette Rincón-Molina, Víctor Manuel Ruíz-Valdiviezo, Rocío Meza-Gordillo, Juan José Villalobos-Maldonado, Miguel Ángel Vences-Guzmán, Reiner Rincón-Rosales

**Affiliations:** 1 Tecnológico Nacional de México/IT de Tuxtla Gutiérrez, Carretera Panamericana Km, 1080, CP 29050 Tuxtla Gutiérrez, Chiapas Mexico; 2 Centro de Ciencias Genómicas, Universidad Nacional Autónoma de México, Av, Universidad s/n, Col. Chamilpa, 62210 Cuernavaca, Morelos Mexico

**Keywords:** Bacterial diversity, Aroclor, Contaminated soils, Polychlorinated biphenyls

## Abstract

Persistent organic pollutants (POPs) such as polychlorinated biphenyls (PCBs) are a group of high-risk synthetic substances for human and environmental health. Currently, the study of sites contaminated by the spillage of equipment PCBs containing have been considered targeted areas for the study of bacterial communities with potential for PCBs degradation. There in isolation of bacterial strains is vital for use in biodegradable processes, such as bacterial bioaugmentation, which accelerates the development of phenomena such as natural attenuation of contaminated sites. The objective of this study was to assess biodiversity of bacteria contained in anthropogenic contaminated soils (H_S_ and H_P_) with PCBs compared to a control sample without contaminant and the modified forest (F) and agricultural (A) soil in the laboratory with 100 mg L^−1^ PCB. For the analysis of 16S rRNA genes amplified from DNA extracted from the soils evaluated, the latest generation of Illumina Miseq and Sanger sequencing for the cultivable strains were detected. The bacteria identified as the most abundant bacterial phyla for H_S_ and H_P_ soil was *Proteobacteria* (56.7%) and *Firmicutes* (22.9%), which decreased in F and A soils. The most abundant bacterial genera were *Burkholderia, Bacillus, Acinetobacter, Comamonas* and *Cupriavidus.* Several species identified in this study, such as *Bacillus cereus*, *Burkholderia cepacia*, *Comamonas testosteroni* and *Acinetobacter pittii* have been reported as PCBs degraders. Finally, by means of a principal component analysis (PCA), a correlation between the physical and chemical characteristics of the soils in relation to the relative abundances of the bacteria identified was obtained. The C/N ratio was directly related to the control soil (without contaminant), while SOM maintained a relationship with F and A soils and the bacterial abundances were directly related to Hs and Hp soils due to the presence of aroclor 1260. Bacteria with the ability to tolerate high concentrations of this pollutant are considered for future use in biostimulation and bioaugmentation processes in contaminated soils.

## Key points

- In this work, the bacterial communities of an anthropogenic site contaminated with polychlorinated biphenyls (PCBs) were analyzed in comparison with the communities detected in forest and agricultural soil evaluated in microcosms contaminated with PCBs.
- A collection of bacterial strains with the ability to tolerate PCBs in culture media was obtained.
- The bacterial communities were related to the parameters N, C and P of the soils that influence bacterial metabolic processes with the presence of PCBs and possibly in the mineralization of the contaminant”.

## Introduction

Polychlorinated biphenyls (PCBs) form a family of 209 congeners characterized by their chemical properties for use in industrial and commercial activities, such as electrical equipment (Furukawa and Fujihara 2008). PCBs are produced as complex mixtures that contained high variability of congeners and not as individual compounds. Each congener is structured by a biphenyl group to which they are attached between one and ten chlorine atoms, depending on the number of chlorine atoms (1–10) and their position (*ortho*, *meta* and *para*), PCB congeners differ in their physical and chemical properties (Passatore et al. 2014). Aroclor is the commercial name of a set of complex mixtures of PCBs (1242, 1254 and 1260) produced and sold in the US, which contains more than 100 PCB congeners. Aroclor is, therefore, the main source of accumulation of PCBs in America continent, including Mexico (Hu et al. 2011). PCBs mixtures are used mostly in the electrical industry, in equipment such as thermostats, condensers and in light transformers, where they constitute the main component (> 70%) of the oils used in this equipment as thermal insulators. These oils are called askareles and are composed of synthetic fluid, chlorobenzoates and PCBs (Hu and Hornbuckle 2010). However, they have high toxicity in humans with immunotoxin effects, tumorogenicity and carcinogenicity capabilities (Hayes et al. 1985; Silberhorn et al. 1990; Tryphonas et al. 1991) and other effects on different organisms (Sager and Girard 1994; Birnbaum 1995; Goldey et al. 1995). PCBs have been banned since 1970 (Matturro et al. 2016) however they are continuously released to the environment through spills, equipment leaks, improper disposal and storage, which represents a serious damage for ecosystems and human health (Nogales et al. 2011; Di Lenola et al. 2018). Therefore, most natural ecosystems and living organisms have been exposed to PCBs for several decades (Tehrani and Van 2014), so the elimination of PCBs accumulated in ecosystems have been an environmental problem at a global level, this challenge pursues the search for biological processes for their elimination (Robertson and Hansen 2015). Thus, these should include naturally occurring biodegradation processes together with added microbial communities able to survive in the presence of such toxic compounds (Matturro et al. 2015). Currently, the identity and role of microorganisms from sites affected by PCBs have been mainly from contaminated marine sediments (Pachiadaki et al. 2010; Pop et al. 2015; Dudášová et al. 2016) where several bacterial enrichments have been obtained and although they have been used matrices contaminated by PCBs, their role is not well documented (Nuzzo et al. 2017). Knowledge on the biodiversity of bacteria associated with sites contaminated by PCBs is still limited, particularly at sites other than marine sediments (Quero et al. 2015; Jugder et al. 2016). But there is ongoing research aimed at exploring the microbiome of sites contaminated with PCBs. *Proteobacteria*, *Acidobacteria* and *Firmicutes* are mainly reported as bacterial phyla associated with PCBs contaminated sediments (Hu et al. 2011; Nuzzo et al. 2017). *Burkholderia*, *Comamonas*, *Cupriavidus*, *Pseudomonas, Rhodococcus* and *Paenibacillus*, among others, have been reported as bacterial genera capable of eliminating certain congeners of PCBs (Qiu et al. 2015; Matturro et al. 2016; Horváthová et al. 2018). The aims of this study were investigate the diversity and abundance of bacteria in soils contaminated with polychlorinated biphenyls and correlate the characteristics of the soil and bacterial communities.

## Materials and methods

### Experimental site description

The experimental site named “La Herradura”, was chosen for the high accumulation of askarels (PCBs congeners) used in electric transformers. This site is located in the municipality of Raudales Malpaso, Chiapas (Mexico) (17.11° N, 93.36° W) at 136 masl, with an annual temperature above 35 °C and average yearly rainfall ~309 mm. The clay soil at the experimental site is classified as Albic Endostagnic Luvisol (Soil Survey Staff 2010).

### Sampling

Askarel oil contaminated soil samples were collected from two sites. A one of them from the surface (Hs) and another 50 cm depth (Hp). Also, a control soil sample (not contaminated by askarel oil) was collected at a distance of 100 m from the Hs and Hp sites. Soil samples were grouped into three composed samples for each site (n = 3). Samples were transferred to sterile tubes of 50 mL. All samples were kept at 8–10 °C during transport to laboratory. Samples used for metagenomic analysis were stored at − 80 °C and those for isolating bacteria in cultures were processed as soon as possible. Additionally, soil samples were collected from a tropical forest and also from an agricultural crop (Additional file 1). Both samples were placed in microcosm and were each were subsequent contaminated with 100 mg L^−1^ askarel oil for 90 days (as a source of PCBs) to compare the effect of the pollutant on in the bacterial communities of these microcosms and correlate them with the control, Hs and Hp soils. The askarel used for samples F and A contained a mixture of aroclor 1242 and 1254 at a concentration of 30 mg L^−1^ and aroclor 1260 to 40 mg L^−1^). These soils were used as well in genomic and metagenomic studies (Matturro et al. 2016).

### Physicochemical analysis of soil

The soils physicochemical properties collected at the Hs and Hp sites contaminated by PCBs (askarel) and of the control soil (uncontaminated) were determined. The pH and electric conductivity (EC) were measured using a digital pH meter Mettler Toledo^®^ Model S220 (New York, USA) in 1:10 (weight/volume) aqueous solution. The soil organic matter (SOM) content, total carbon and C:N ratio were analyzed according to AOAC methods (AOAC 1996). Total nitrogen was measured by Kjeldhal method (Bremner 1996). Total phosphorus was determined with the solubilization method of HNO_3_/HClO_4_. Also, the same determinations were done for forest (F) and agricultural (A) soil samples that were previously contaminated with askarel at 100 mg L^−1^.

### Polychlorinated biphenyl (PCB) quantification

The concentration of polychlorinated biphenyl (PCB) in soil samples that were collected at the control, Hs and Hp sites and from the collected slurry in each microcosm contaminated by askarel oil was determined in terms of aroclor 1242, aroclor 1254 and aroclor 1260 as follows: The PCBs were extracted from 5 g of soil with 20 mL of pentane HPLC grade (Sigma-Aldrich, USA) and then mixed in vortex for 5 min. Next, the supernatant was placed in a 15-mL Falcon tube and the procedure was repeated twice. The pentane was then concentrated to 1.0 mL using a rotary evaporator. Analysis was performed by gas chromatography (GC) with electron capture detector (ECD) on a Trace GC equipped with a 30-m × 0.25-mm × 0.25-μm HP5 capillary column (Agilent Technologies, Palo alto, CA, USA). The injector and transfer line temperatures were 250 and 300 °C, respectively. The oven temperature was held at 60 °C for 1 min, then increased to 160 °C at a rate of 20 °C min^−1^, further increased to 300 °C at a rate of 6 °C min^−1^, and held for 2 min. helium was employed as the carrier gas with a constant flow of 1 mL min^−1^. For quantitative analysis, the PCB calibration mixes in aroclor 1242, 1254 and 1260 (Sigma-Aldrich, USA) were used.

### DNA extraction and PCR amplification of bacterial 16S rRNA gene

DNA was extracted from 1.5 g soil (three times from 0.5 g) through the DNeasy Power Soil commercial kit. DNA concentration was quantified on a Nano Drop 2000 spectrophotometer. Triplicate PCR reactions were done to amplify the V3-V4 16S rRNA gene hypervariable regions for each metagenomic DNA sample. PCR amplification were done using 8-pb barcoded primers 341-F (5′-CTACGGGGGCGCAG-3′) and 805-R (5′-GACTACGGGTATCTAATCC-3′). Pool PCR products were clean using FastGene columns (Nippon Genetics, Co., Ltd) and amplicon products quantification was done with Nanodrop and then sequencing was performed by Macrogen Inc. (DNA Sequencing Service, Seoul, Korea) using Illumina Miseq 2 × 300 paired-end (Ceja-Navarro et al. 2010).

### Analysis of genetic sequences

The QIIME version 2.0 software pipeline was used to analyze the sequencing data (Caporaso et al. 2010a). The poor quality readings were eliminated from the data sets, i.e. quality score < 25, containing homopolymers > 6, length < 400 nt, and containing errors in primers and barcodes. Operational taxonomic units (OTUs) were determined at 97% similarity level with UCLUST algorithm (Edgar 2010). Chimeras were detected and removed from the data sets using the Chimera Slayer (Haas et al. 2011). Sequence alignments were done against the Greengenes core set and using representative sequences of each OTU using PyNAST, and filtered at a threshold of 75% (Caporaso et al. 2010b). Taxonomic assignation was done with rarified data sets at 850 reads per sample to compare the same amount of sequences and using the naïve Bayesian rRNA classifier from the Ribosomal Data Project (http://rdp.cme.msu.edu/classifier/classifier.jsp) at a confidence threshold of 80% (Wang et al. 2007). The illumina sequencing data reported herein was registered in the NCBI as a BioProject (ID: PRJNA622403) and deposited as a Sequence Read Archive (SRA) database under accession numbers of SAMN14514156 to SAMN14514160.

### Bacterial isolation and DNA extraction

Five g of each soil was placed in modified minimum medium (8.1% (NH_4_)_2_SO_4_, 16.31% K_2_HPO_4_, 4.91% NaCl and MgSO_4_•7H_2_O 49.18% dextrose anhydride, 16.11% yeast extract and 0.49% FeSO_4_•7H_2_O) added with 250 mg L^−1^ of biphenyl. For this initial process, soils were placed in 25 mL broth obtaining a base solution. Then serial dilutions 10^−1^ to 10^−6^ were done and 10 µL of each bacterial dilution was streaked on the minimum medium previously mentioned. Plates were incubated at 30 °C for 5 days. Pure cultures were preserved in 65% glycerol-minimum medium broth at 4 °C. Total genomic DNA of each strain was extracted using the DNA Isolation Kit Fungal/Bacterial (Zymo Research) according to the manufacturer specifications. Extracted genomic DNA was verified by 1% agarose gel electrophoresis and with Nanodrop’.

## 16S rDNA gene, genetic fingerprinting and phylogenetic analysis of the isolates

To generate DNA genetic patterns BOX_A1R oligo was used, as described by Koeuth et al. (1995). The genomic patterns were identified through electrophoresis in 1.5% agarose gels. The Shannon–Weaver index of richness (d) and diversity (H) were calculated based on BOX_PCR genetic profiles. The PCR of the 16S rDNA gene was performed with the universal primers for bacteria fD1 (5′-AGAGTTTGATCCTGGCTCAG-3′) and rD1 (5′-AAGGAGGTGATCCAGCC-3′) (Weisburg et al. 1991) using an Applied Biosystems model 2720 thermocycler (Ca, USA). PCR conditions consisted of an initial denaturing step at 94 °C for 5 min, 35 cycles (94 °C for 1 min, 55 °C for 1 min and 72 °C for 2 min) and an additional final chain elongation step at 72 °C for 7 min. The size of the amplification products was verified by electrophoresis in 1% agarose gels. PCR products were purified using the PCR product purification system kit (Roche TM, Switzerland). The PCR-16S rDNA products were digested with the RsaI restriction enzyme (Thermo Scientific) using the Amplified rDNA Restriction Analysis (ARDRA) and were observed through electrophoresis in 3% agarose gel, to use them in diversity analysis. The amplification mixture was purified using the Roche PCR product purification system, before sequencing. The PCR products sequenced (Macrogen), were compared using BLAST and analysis tools of Ribosomal Database Project-II (Altschul et al. 1990). The taxonomically related sequences obtained from the National Center for Biotechnology Information (NCBI) were aligned by the CLUSTAL X (2.0) software with default settings (Larkin et al. 2007). Phylogenetic and molecular evolutionary analysis were performed with MEGA v5.2 (Tamura et al. 2011).

### Nucleotide sequence accession numbers

The sequences of strain reported herein were deposited in the GenBank with the accession numbers from MH921878, MH921879, MH921881, MH921883, MH921884, MH921887 and MH921888 for strains isolated from control soil. For the isolates obtained from the Hs site, the range of numbers were from MH209072 to MH209076. The range of numbers for the Hp site were from MH921875 to MH921877. The sequences for the isolates of forest soil (F) contaminated with PCBs were registered with the numbers MN685207, MN685208, MN685209, MN818571 and MN818574, and those isolated from the agricultural soil (A), the numbers were MN685210, MN685211, MN685212, MN818572 and MN818573.

In the case of the bacterial strains (Table 4), these were deposited in the collection of microbial strains of the ‘Centro de Ciencias Genomicas’ CCG-UNAM (Mexico).

### Statistical analysis

Physicochemical variables of soils and concentration of PCBs (as aroclor congeners mixtures) were evaluated by one-way analysis of variance (ANOVA). Mean difference significance was tested with the Tukey test and also by the *t*_student’s statistics (*p *< 0.05). The correlation between relative abundance of the bacterial groups at phyla taxonomic level and each type of soil, and the relation of the abundance with physicochemical characteristics were explored with a principal component analysis (PCA). Minitab 18.1 was used for statistics analyses (2017 Minitab, Inc. All rights reserved).

## Results

### Soil characteristics

The physicochemical analysis allowed to determine that there are significant variations between the different parameters evaluated in anthropogenic soils contaminated with PCBs compared to agricultural (A) and forest (F) soils that were enriched with the pollutant askarel (Table 1). In the case of pH, this proved to be more acidic in soils collected at sites Hs and Hp, where there was a high concentration of PCBs, while in forest soils and in agricultural soils, the pH was slightly alkaline. In the case of electric conductivity (EC) did not show significant difference in any of the samples evaluated. The apparent density was different among the treatments evaluated (soils), being lower in those soils that presented anthropogenic contamination by askarels. With respect to the parameters related to soil fertility (SOM, total carbon, total nitrogen and total phosphorus) showed significant differences (*p *< 0.05) between the treatments evaluated. The soil organic matter (SOM) content was significantly higher in soils of agricultural nature contaminated with PCB compared to the rest of the treatments. Also, the total carbon, total nitrogen and total phosphorus content was higher in this type of soil. The C:N ratio is a parameter that indicates the functionality of the soils. In our case, control soil samples (without PCBs) had a higher C:N ratio compared to PCB contaminated soils.

**Table 1:** Characteristics of soil contaminated PCBs

Sample	Soil classification (texture)^a^	pH	EC (dS m^−1^)	Density (g mL^−1^)	SOM (%)	Total P (mg/kg)	Total C (mg/kg)	Total N (mg/kg)	C:N ratio
Control	Luvisol (clayey)	5.5 E^b^	0.063 A	1.17 B	14.17 C	5.65 C	7.26 BC	0.53 C	13.70 A
H_S_		5.8 C	0.047 A	1.0 D	10.73 D	4.85 D	6.40 CD	0.48 D	11.75 B
H_P_		5.6 D	0.063 A	1.08 C	9.04 E	6.06 B	4.84 D	0.49 D	9.81 D
F	Regosol (silty–loam)	7.0 B	0.076 A	1.25 A	20.11 B	3.48 E	9.05 AB	0.91 B	9.87 D
A		7.8 A	0.055 A	1.15 B	21.46 A	10.44 A	10.65 A	0.96 A	11.09 C
*p*-value HSD (*p* < 0.05)		0.0000	0.6824	0.0000	0.0000	0.0000	0.0000	0.0000	0.0000
		0.0404	0.0663	0.0416	0.1953	0.0628	1.7888	0.0360	0.6158

*EC* electric conductivity, *SOM* soil organic matter, *C:N* carbon:nitrogen ratio, *HSD* honest significant difference (Tukey’s test)

^a^According to Soil Survey Staff (2010)

^b^The means followed by the same capital letter do not show any significant differences (Tukey’s test, *p* < 0.05)

The content of PCBs in terms of mixtures of aroclors 1242, 1254 and 1260 was determined in polluted soils collected at sites Hs, Hp, F and A (Table 2). Gas chromatography allowed to determine that the concentration of the mixture of aroclor 1242 was higher in the soils obtained from the Hs site (30.72 mg kg^−1^) and in the case of the mixture of aroclor 1254 it was detected in a lower concentration (< 10.54 mg kg^−1^), however, a higher concentration of this contaminant (> 60 mg kg^−1^) was recorded in the aroclor 1260 mixture. On the other hand, the total sum of the concentrations of the three mixtures of aroclor (1242, 1254 and 1260) was higher at the Hp site (122.39 mg kg^−1^) compared to the aroclor concentration determined at the Hs site (101.98 mg kg^−1^). These concentrations of PCBs were detected in the samples are above the permitted values according to the NOM-133-SEMARNAT-2015 standard for soils contaminated by PCBs in Mexico. In the case of soil samples F and A, a concentration of 100 mg kg^−1^ of the aroclor mixture was detected.

**Table 2:** Quantification of polychlorinated biphenyls (PCBs) as aroclor mixture in soil sample

Sample	Aroclor (mg kg^−1^)			
	1242	1254	1260	Total mixture
Control	ND	ND	ND	ND
H_S_	30.72 ± 2.6	10.54 ± 1.6	60.72 ± 6.2	101.98 ± 10.4
H_P_	25.45 ± 3.2	8.75 ± 1.2	88.19 ± 7.6	122.39 ± 12.0
F	30.0 ± 0.1	30.0 ± 0.1	40.0 ± 0.1	100.0 ± 0.1
A	30.0 ± 0.1	30.0 ± 0.1	40.0 ± 0.1	100.0 ± 0.1

*ND* not detected

### Bacterial communities structure in the soils

A total of 14,500 sequences were obtained of all the soil samples with the observed OTUs (Operational Taxonomic Units) ranging from 1751 to 4489 for the five soils evaluated.

Richness and diversity of bacterial species were estimated in the different soil samples using cultivable and non-cultivable methods showing significant variations (Table 3). In the non-cultivable method, both the Chao index as well as the ACE showed a high abundance (*d*) of bacterial species in the Hp soil that were contaminated with PCBs (aroclor mixture). Likewise, the diversity (*H*) of species were greater in the Hp soil according to the Shannon and Simpson index. In contrast to the cultivable method, the Shannon–Weaver index estimated a high abundance (*H* = 4.7) and diversity (*d* = 1.3) of species in the control soil (without contaminant). Both the abundance and diversity of species are decreasing as soils have been contaminated by PCBs.

**Table 3:** Richness and diversity of bacterial communities in soils contaminated PCBs

Sample	OTUs	Index					
		Non-cultivable method				Cultivable method	
		Richness (*d*)		Diversity (*H*)		Richness (*d*)	Diversity (*H*)
		Chao 1	ACE	Shannon	Simpson	Shannon–Weaver	
Control	3345	6252.3	7076.8	9.3	1.0	4.7	1.3
H_s_	3063	6013.7	6551	8.6	1.0	4.1	1.1
H_p_	4489	9425.7	10,474.1	10	1.1	3.2	0.9
F	1751	1979	2117.4	7.8	0.9	3.4	1.0
A	1852	2261.4	2492.1	7.5	0.9	3.3	1.0

*ACE* abundance-based coverage estimator

OTU: Operational taxonomic units as determined by Uclust (Edgar 2010) at a similarity threshold of 97%

**Fig. 1:**
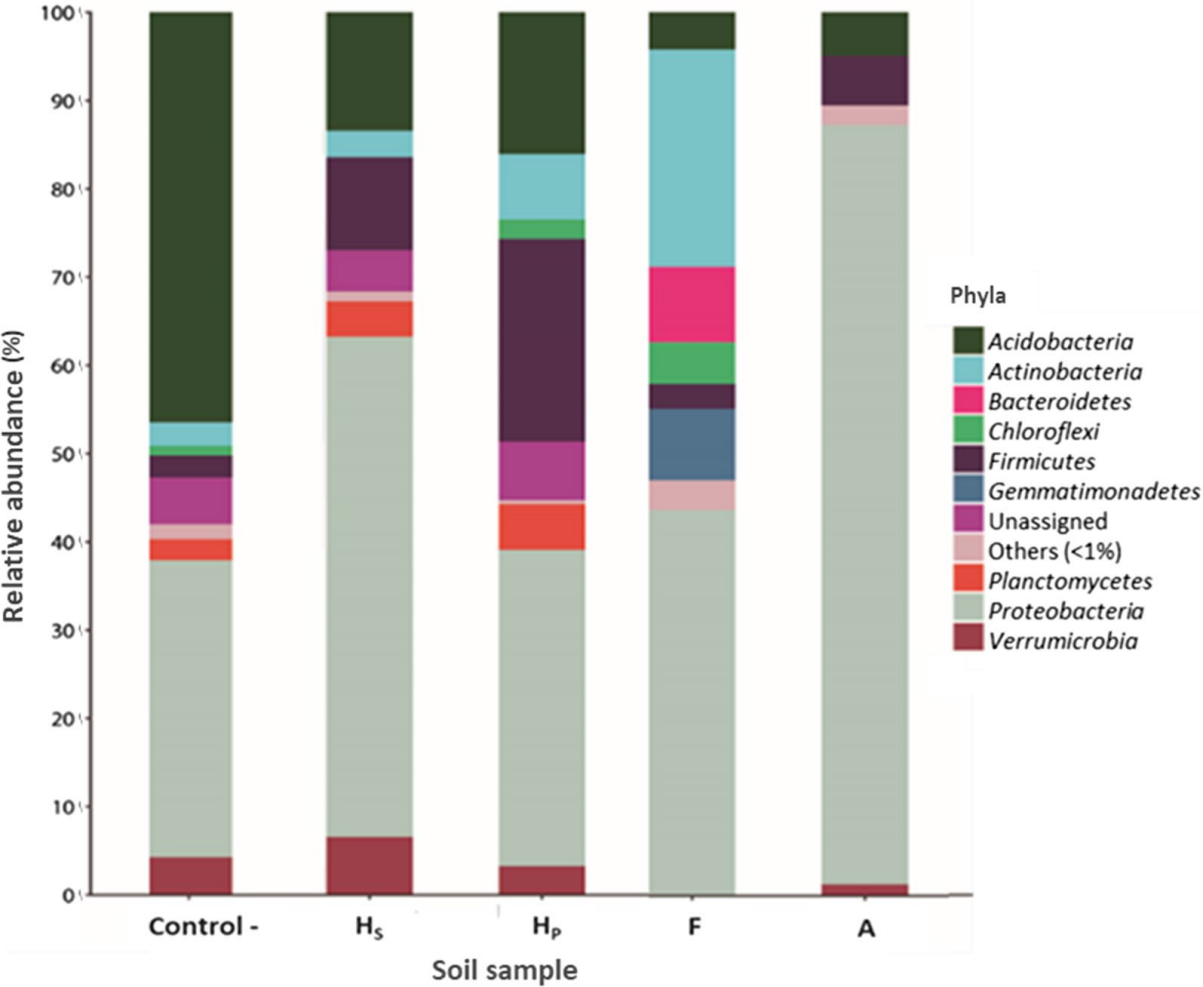
Bar plot with relative abundance of the different bacterial phyla found in soils contaminated PCBs

The identification of bacterial phyla was over 90%, in the different soils evaluated (Fig. 1). Phylotypes belonged to 9 different phyla, which contributed >1% of the sequences. The control sample (without PCBs) showed *Acidobacteria* (46.4%), *Proteobacteria* (33.7%), *Verrucomicrobia* (4.3%), *Actinobacteria* (2.7%) and *Firmicutes* (2.5%). The soil Hs sample contaminated with PCBs (aroclor mixture) showed a greater abundance of *Proteobacteria* (56.7%), *Firmicutes* (10.5%), *Actinobacteria* (3.0%) and *Verrucomicrobia* (6.6%), but a marked decrease of *Acidobacteria* (13.4%). In the case of the sample H_P_ also showed an increase of *Proteobacteria* (23.0%), *Acidobacteria* (16.0%), *Firmicutes* (23.0%), *Actinobacteria* (7.4%) and *Verrucomicrobia* (3.3%).

With respect to the soil samples obtained from F and A site, previously contaminated *in vitro* with PCBs (aroclor mixture) showed significant variations in relation to the relative sequence abundances at the phyla level. In sample F the relative abundance of the phyla *Actinobacteria* and *Proteobacteria* increased significantly (24.6 and 43.7%, respectively), but showed a decrease in *Acidobacteria* (4.2%). Sample A showed an increase in *Proteobacteria* (86.0%) and *Firmicutes* (5.6%), but had a decrease in relative abundance in *Acidobacteria* (4.9%).

**Fig. 2:**
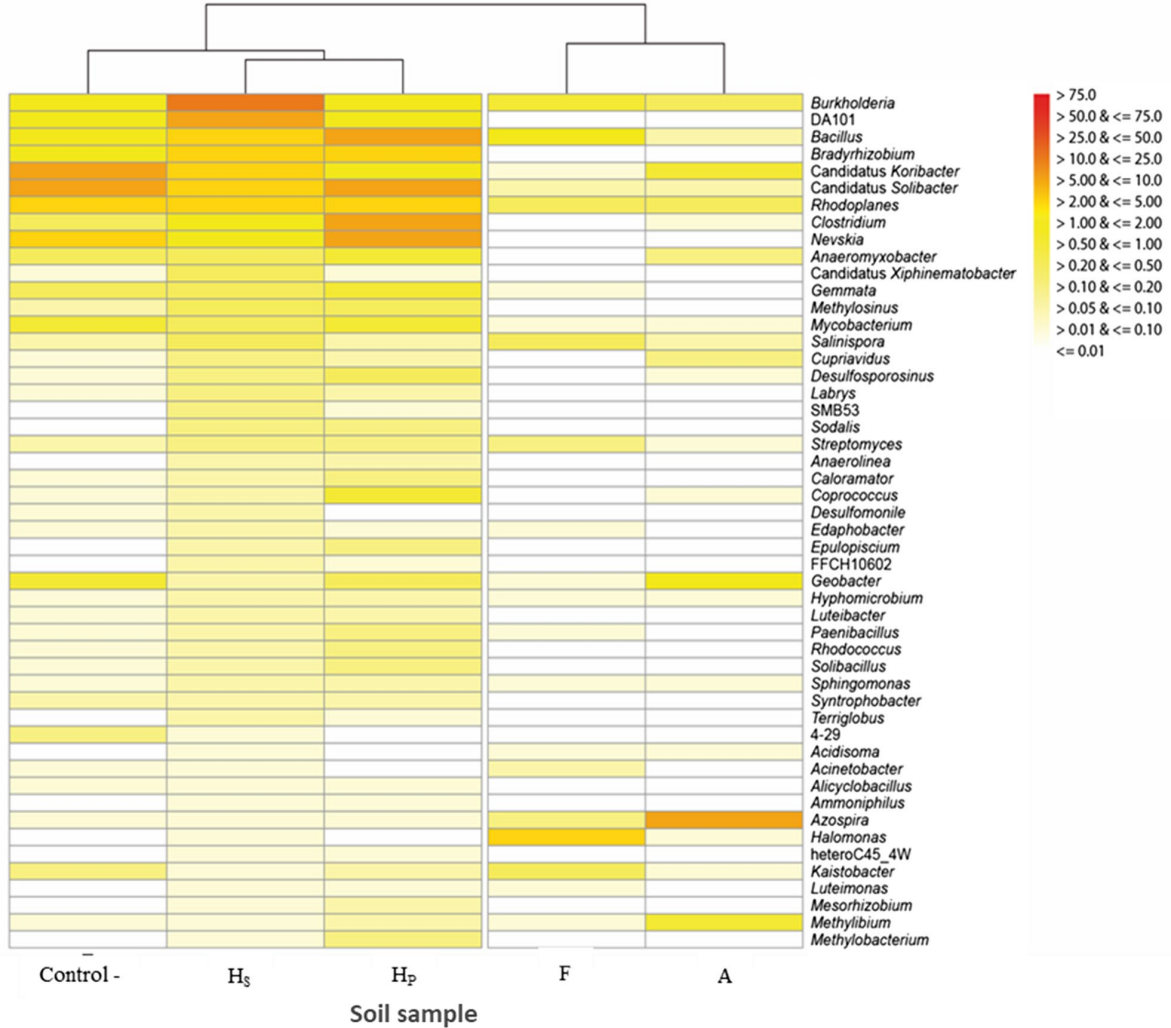
Heatmap with the relative abundance of different bacterial genera found in soils contaminated PCBs

The bacterial composition of the control, Hs, Hp, F and A soils was studied at the genera level (Fig. 2). Fifty bacterial genera were identified. In the control sample (without contaminant), *Candidatus solibacter* belonging to the *Acidobacteria* phylum was the main genus found with a relative abundance >5%. Also, bacteria belonging to the genera *Burkholderia*, *Bacillus*, *Clostridium*, DA101, *Rhodoplanes* and *Mycobacterium* with a relative abundance >1% were detected. In the case of the Hs sample (collected from PCB contaminated soil), *Burkholderia* belonging to *Betaproteobacteria* phylum was the most abundant bacterial genus> 18%. Also, DA101 was among the main phylotypes found in this soil with a relative abundance >5%. In contrast, the *Bacillus*, *Candidatus koribacter*, *Rhodoplanes* and *Clostridium* genera showed a low abundance >1.0%. In the soil sample collected at the Hp site, the genera *Bacillus*, *Candidatus*, *Clostridium* and *Nevskia* were identified with a relative abundance higher than 6.0%. *Burkholderia*, DA101, *Bradyrhizobium* and *Rhodoplanes* with a relative abundance >1.0% were also the bacterial genera found in this type of soil contaminated by PCBs (aroclor mixture).

With respect to F and A samples that were previously contaminated *in vitro* with a mixture of aroclor, these showed significant variations in relation to the relative abundance of the bacterial genera identified in these soils. The bacterial genera *Burkholderia*, *Bacillus*, *Halomonas*, *Kaistobacter*, *Rhodoplanes*, and *Streptomyces* were identified in sample F with abundance above 1%. While the genus *Azospira* with an abundance >8.0%, as well as the genera *Burkholderia*, *Rhodoplanes* and *Cupriavidus* with an abundance >1.0% were identified as bacterial components in soil sample A.

A phylogenetic analysis based on the 16S rRNA gene sequences of cultivable bacterial strains isolated from different PCB-contaminated soils was performed. A total of 135 bacterial strains were obtained from different soil sampling sites (Control, H_S_, H_P_, F and A). Thus, of the 135 strains isolated, 25 different ARDRA genomic profiles were obtained (Table 4). Of which seven morphotypes corresponded to the control soil, five were from the soil sample Hs, three from the sample Hp. Also five ARDRA profiles were of samples obtained from forest soil and five corresponded to agricultural soils. The Shannon–Weaver index showed a higher diversity (*H *= 1.3) and richness (*d* = 4.7) of bacterial species isolated from the control sample, in contrast to the diversity of bacterial species in soils contaminated with PCBs (aroclor), which decreases significantly.

**Table 4:** Taxonomic affiliation of the bacteria isolated of soils contaminated PCBs

Sample site	ARDRA profiles^a^	Phylogenetic relationship			Phylum
		Representative isolate	Closest NCBI match/similarity (%)^b^	Accession number	
Control soil	A-A	DCB13	*Bacillus thuringiensis* L2.TYA/95.6	MH921878	*Firmicutes*
	A-B	DCB14	*Bacillus cereus* H3/96.4	MH921879	*Firmicutes*
	A-C	DCB18	*Bacillus paramycoides* SBMS4/95.7	MH921881	*Firmicutes*
	A-D	DCB20	*Achromobacter denitrificans* DBT224/98.9	MH921883	*Proteobacteria*
	A-E	DCB21	*Bacillus paranthracis* J-131/98.8	MH921884	*Firmicutes*
	A-F	DCB26	*Cupriavidus malaysiensis* USMAA1020/99.2	MH921887	*Proteobacteria*
	A-G	DCB27	*Bacillus cereus* XS 2-8/97.7	MH921888	*Proteobacteria*
Hs	B-A	DCB01	*Burkholderia cenocepacia* Z6/96.0	MH209072	*Proteobacteria*
	B-B	DCB02	*Burkholderia ambifaria* ChDC B361/95.2	MH209073	*Proteobacteria*
	B-C	DCB03	*Burkholderia cepacia* BC16/95.3	MH209074	*Proteobacteria*
	B-D	DCB04	*Myroides odoratus* (LT899994.1)/96.6	MH209075	*Flavobacteriia*
	B-E	DCB05	*Bacillus cereus* PR12/97.0	MH209076	*Firmicutes*
Hp	C-A	DCB07	*Burkholderia anthina* MYSP113/97.0	MH921875	*Proteobacteria*
	C-B	DCB08	*Burkholderia vietnamiensis* BU97/95.0	MH921876	*Proteobacteria*
	C-C	DCB12	*Bacillus anthracis* A4/98.3	MH921877	*Firmicutes*
F	D-A	DCB101	*Acinetobacter pittii* AB17H194/99.0	MN685207	*Proteobacteria*
	D-B	DCB102	*Kosakonia arachidis* LGR-9 /99.5	MN685208	*Proteobacteria*
	D-C	DCB103	*Comamonas testosteroni* OTU-c14/98.1	MN685209	*Proteobacteria*
	D-D	DCB120	*Enterobacter ludwigii* 7D2C3/99.7	MN818571	*Proteobacteria*
	D-E	DCB124	*Staphylococcus saprophyticus* SPB40-5/99.4	MN818574	*Firmicutes*
A	E-A	DCB104	*Acinetobacter baumannii* B8342/99.0	MN685210	*Proteobacteria*
	E-B	DCB105	*Burkholderia cenocepacia* Z6/97.6	MN685211	*Proteobacteria*
	E-C	DCB106	*Comamonas testosteroni* F4/98.6	MN685212	*Proteobacteria*
	E-D	DCB122	*Enterobacter oryzae* Ola 01/98.1	MN818572	*Proteobacteria*
	E-E	DCB123	*Comamonas testosteroni* 22/99.9	MN818573	*Proteobacteria*

^a^ Representative isolate selected by ARDRA profile

^b^ Similarity percentage was estimated by considering the number of nucleotide-substitutions between a pair of sequences divided by the total number of compared bases × 100%

The isolates were taxonomically classified within the phyla *Proteobacteria*, *Firmicutes* and *Flavobacteria* (Table 4). In detail, the bacterial genera *Achromobacter*, *Bacillus* and *Cupriavidus* were identified from the control sample (A-ARDRA group). For the H_S_ sample (B-ARDRA group), the genera *Burkholderia*, *Bacillus* and *Myroides* were the most abundant. In the case of H_P_ sample (C-ARDRA group), *Burkholderia* and *Bacillus* were the genera grouped. *Acinetobacter, Comamonas, Enterobacter, Kosakonia* and *Staphylococcus* were identified in F sample (D-ARDRA group). Finally, the isolates obtained the A sample (E-ARDRA group) were grouped within the genera *Acinetobacter, Burkholderia, Comamonas* and *Enterobacter.*

### Principal component analysis (PCA)

**Fig. 3:**
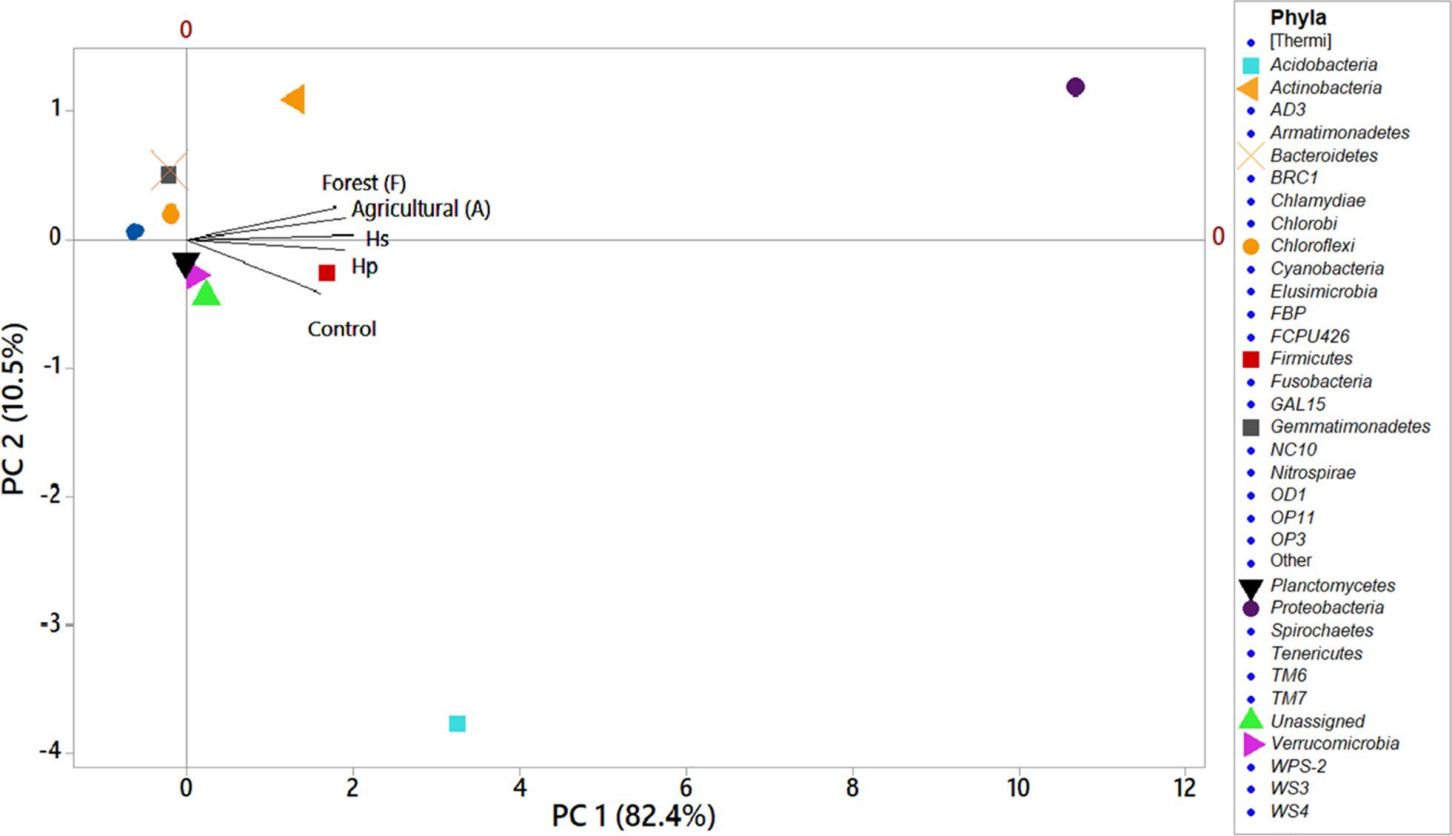
The principal component analysis separated the relative abundances of the different bacterial phyla found in the sites contaminated by PCBs, both anthropogenic and comparative control, in relation to the types of soil evaluated (Control, H_S_, H_P_, F and A). The main component (PC) 1 varies 82.4% of the variation and (PC) 2 (10.5%)

**Fig. 4:**
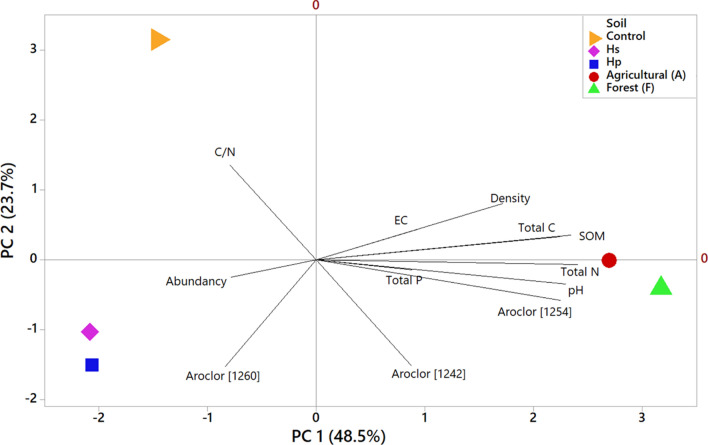
Principal component analysis considering the relative abundance of the different bacterial phyla, soil characteristics and levels of PCBs contamination. Principal component (PC 1) explained 48.5% of the variation and PC 2 (23.7%)

The PCA considered the relative abundances of the different bacterial phyla and the separated (H_S_ and H_P_) anthropogenic samples contaminated with PCBs and their control (without PCB) from the comparative samples of forest (F) and agricultural (A) (Fig. 3). The total soil samples evaluated were characterized by a positive PC1, where the bacterial phyla with the greatest relative abundance were *Actinobacteria*, *Firmicutes*, *Verrucomicrobia* and *Plantomycetes*, which are considered oligotrophic organisms. While the bacterial phyla *Gemmatimonadetes* and *Chloroflexi* presented negative PC1 and were characterized by greater relative abundance. The samples of F, A and H_S_ soils were characterized by a positive PC2 related to the group of phyla *Actinobacteria*, *Gemmatimonadetes* and *Cloroflexi*, which have been related in processes of remediation of contaminants, while the phyla *Firmicutes*, *Verrucomicrobia* and *Plantomycetes* were related to H_P_ and control soils by a negative PC2. The rest of the evaluated phyla were located at the same point within the PCA, located with a negative PC1 and positive PC2, but their relative abundance was relatively small for their separation. Another PCA related the specific abundances of bacterial phylum with the physicochemical characteristics of the soil and the PCBs concentrations in aroclor mixtures quantified in the different samples (Fig. 4). F and A samples were characterized by a positive PC1 in counterpart with the control. H_S_ and H_P_ samples that were characterized by a negative PC1.

With regard to physicochemical characteristics, pH, EC, density, total C, total P and SOM were characterized by a positive PC1, while total N was the only property that had a negative PC1. With respect to the concentrations of PCBs, mixtures of aroclor 1242 and 1254 had a positive PC1 in counterpart with the mixture of aroclor 1260 which presented a negative PC1. The EC, density, total C and SOM properties were characterized by a positive PC2, in contrast to the pH, Total P, total N and the three mixtures of aroclor that had a negative PC2.

## Discussion

The polychlorinated biphenyls (PCBs) contamination in ecosystems and terrestrial biomes around the world continues to worry to scientists, considering the high degree of toxicity of this compound. For this reason, the search and selection of native microorganisms with potential of the removal and degradation of PCBs is increasing significantly. In this study, the structure and diversity of native bacterial communities in soils contaminated with PCBs were evaluated. The physical–chemical analysis of the soils collected at contaminated sites (Hs, Hp, F and A) and the control sample (uncontaminated) was evaluated, while the data analysis allowed to determine significant differences between the sampling sites (treatments) in relation to the studied parameters (Table 1). For instance, the soil pH was more acidic in the control samples and also in those contaminated samples (Hs and Hp) compared to the F and A samples, which had a neutral pH (7.0 to 7.8). There is no clear information that PCBs has an effect on soil pH. The pH variations recorded in the soils can be attributed to the geological nature of the soil. That is, the control, Hs and Hp soils were classified as luvisol (Soil Survey Staff 2010). These soils are clay and slightly acidic, with an adequate content of organic matter and a high content of aluminum and iron. In the case of F and A soils, these were of the regosol type, which are slightly alkaline, with a high content of organic matter and rich in calcium and magnesium. Likewise, no discernable differences between the different samples were determined in related to electrical conductivity (EC). It is known that the EC is related to the salinity content of the soil (Zenteno-Rojas et al. 2019). The samples obtained in soil F had the highest EC value (0.076 dS m^−1^), this could be due to the high content of Ca^2+^ and Mg^2+^ ions that is common in soils obtained from tropical forests (regosols). The apparent density varied significantly (*p* < 0.05) among the analyzed soils. In soil sample F a high density was recorded, indicating that it is a highly compacted soil. Both characteristics influence the density and water-holding capacity, which facilitates the accumulation of other substances, such as askarel oils in this type of soil (Horváthová et al. 2018).

In F and A soils a high content of soil organic matter (SOM) was found compared to the control, Hs and Hp soils (Table 1). This is important, considering that organic matter and PCBs are the main source of carbon that will be used by bacterial communities to carry out the biochemical processes of mineralization of these organic compounds. The analysis of variance showed that there are significant differences (*p* < 0.05) between the different PCB-contaminated soil samples in relation to content of total C, total N and total P in the different PCB-contaminated soil samples. This phenomenon can be attributed to the metabolic activity of the bacterial communities in the soil that contribute to the mineralization processes (Cervantes-González et al. 2019). Otherwise, the C:N ratio determined in soil was higher in control samples when compared with the rest of the treatments (soil samples). The biochemical processes of decomposition of organic matter and mineralization that occurs in soils directly influenced the C:N ratio. The variability of this parameter can also be attributed to nitrogen mineralization due to microbiological decomposition and the metabolization of PCBs by natural attenuation (Lladó et al. 2017).

In relation to the quantification of polychlorinated biphenyls (PCBs) as aroclor mixture (1242, 1254 and 1260) significant variations in the levels of contamination in the soil samples by each of the PCB congeners were observed (Table 2). The Hs and Hp samples recorded a high concentration of the aroclor compared to the other soil samples analyzed in this study. The concentration was higher in Hp soil possibly because the sample was collected at 50 cm depth where there was a higher concentration of askarel (PCBs congeners). It was also noted that aroclor 1242 had a lower concentration than the 1260 mixture in both the Hs and Hp samples, possibly due to volatilization or lixiviation. Similar results were reported by Jing et al. (2018) where they evaluated the distribution of PCBs in a wastewater effluent for 5 years and found that tri-, tetra-and penta-chlorinated congeners correspond to PCBs with a higher recalcitrant index at the sites of evaluation. Otherwise, the heavier aroclor (such as aroclors 1254 and 1260) are known to be recalcitrant to volatilization, and to aerobic degradation, in natural settings. It has been reported that this type of PCBs congeners persist in contaminated soils despite the exposure of several rain cycles (Kaya et al. 2017). For this reason, the bacterial communities that inhabit these contaminated soils play a very important role in the processes of degradation of the different PCB congeners. Several bioremediation experiments have been carried out and in which the efficiency of bacterial strains in the degradation of recalcitrant toxic compounds, such as PCBs, has been demonstrated. For instance, Hatamian-Zarmi et al. (2009) evaluated the aerobic PCB degradation by *Pseudomonas aeruginosa* TMU56 isolated from soil that had been contaminated with electrical transformer fluid (askarel) for over 35 years. This bacterial strain was capable of decomposing PCB congeners, such as aroclor 1242.

Regarding the diversity and composition of the bacterial community that inhabit the soils contaminated by PCBs, significant variations were observed in relation to the number of OTUs identified (Fig. 1) and also in the different indices of diversity and richness (Table 3) that were estimated. Despite the high concentrations of PCBs congeners (122.39 mg L^−1^ of aroclor mixtures) contained in Hs and Hp soils, a greater number of OTUs were determined (3063 and 4489, respectively) compared to soil samples F and A (contaminated in-vitro with 100 mg L^−1^ of aroclor mixture). In the case of the sample Hp showed a greater relative abundance of *Proteobacteria* (23.0%), *Acidobacteria* (16.0%), *Firmicutes* (23.0%), *Actinobacteria* (7.4%) and *Verrucomicrobia* (3.3%). Each of these phyla group an important diversity of bacterial species with biological features to tolerate and degrade toxic compounds, such as PCBs. Both, the Chao index as well as the ACE confirmed the increasing diversity in the contaminated soil, especially in Hp soil compared to the other samples. It has been detected that persistent organic pollutants (POPs) can accumulate in sediments and soils for a long time and potentially influence on the composition and diversity of bacterial communities (Sun et al. 2012). Then in these contaminated soils xenobiotic become limiting abiotic factors that exert selective pressure on microbial communities, altering the abundance and diversity of bacterial species (Bent et al. 2007). Paissé et al. (2008) studied the structure of bacterial communities along a gradient hydrocarbon contamination in coastal sediment. These authors indicated that bacterial community structure was obviously associated with the gradient of oil contamination. Our results, indicate that chemical contamination reduces bacterial richness and this pattern is in accordance with ecological theories that predict multiple stressors lead to decreased diversity, due to the inability of certain individuals to develop tolerance (Vinebrooke et al. 2004).

Metagenomic analysis based on 16S rRNA gene sequencing allowed the identification of nine different bacterial phyla in the soils evaluated (Fig. 1). Bacterial phylotypes grouped in the phyla *Proteobacteria*, *Firmicutes*, *Actinobacteria* and *Verrucomicrobia* were identified in soil samples Hs and Hp contaminated with PCBs. In these soils, *Proteobacteria* was the phylum that recorded the highest relative abundance. Several of these bacterial phyla have been isolated from different environments and a wide diversity of bacteria affiliated with these phyla have shown high capacity to tolerate and degrade PCBs. As is the case of *Burkholderia xenovorans (*member of *Betaproteobacteria*), a bacterium isolated from an acidic PCB-polluted soil showed high capacity to degrade this recalcitrant toxic chemical compound (Nogales et al. 2001). Also, Aguirre et al. (2007) studied the diversity of bacteria associated with the rhizospheric soil of plants and found that member of *Betaproteobacteria* had a high abundance mainly in soils contaminated with PCBs.

With respect to comparisons made with F and A soils, the most abundant and contrasting bacterial phyla with anthropogenic soils Hs and Hp were *Actinobacteria*, *Bacteroidetes* and *Chloroflexi*. The *Actinobacteria* and *Bacteroidetes* have been identified in marine sediments contaminated by PCBs (Sun et al. 2013). Regarding the dechlorination processes of PCBs, Matturro et al. (2015) evaluated anaerobic processes in sediments contaminated by PCBs where phylum *Chloroflexi* was the one that presented the greatest chlorine removal potential.

The analysis of bacterial community of contaminated soils at the genera level (Fig. 2) showed a greater relative abundance of *Bacillus*, *Burkholderia*, *Candidatus, Clostridium* and *Solibacter* in the anthropogenic Hs y Hp samples compared to F and A samples, where the genera identified had low relative abundance. In the Hs sample, genus *Burkholderia* registered the highest abundance in relation to the other genera identified. In the pioneering studies of bioremediation of contaminated soils, the genus *Burkholderia* has been considered one of the most important in PCB degradation, because it has specific enzymes that catalyze these complex chemical degradation reactions (Agulló et al. 2007). Bartels et al. (1999) identified the expression of the *bphK* gene related to biphenyl metabolism in the genome of the *Burkholderia xenovorans* LB400 strain and later, Denef et al. (2004) analyzed the metabolism of assimilation of chlorobenzoates and biphenyl by the strain LB400 through an outline of metabolic networks. Another important bacterium is the *Bacillus* sp. JF8, which has shown in potential for the degradation of polychlorinated biphenyl and naphthalene (Hatta et al. 2003).

In regard to the taxonomic identity of the bacterial species, the phylogenetic analysis of the 16S rDNA gene sequence showed that the bacterial community isolated from soils contaminated by polychlorinated biphenyls (Table 4) included three major phylogenetic groups (*Proteobacteria*, *Firmicutes* and *Flavobacteria*). These phyla include a wide diversity of bacterial species that have specialized metabolisms, such as phototrophy, photoheterotrophy, and chemilithotrophy that allow them to degrade different persistent organic pollutants (POPs), including PCB congeners (Sun et al. 2013; Matturro et al. 2015; Mikolasch et al. 2019). The isolates from control soil were grouped within seven different ARDRA genomic profiles (A-A to A–G). The species grouped into the genus *Bacillus*, *Achromobacte*r and *Cupriavidus.* The Genus *Bacillus* (Phylum *Firmicutes*) was the most abundant in this type of soil. These bacteria are metabolically versatile, chemolithotrophy and form spores as a survival strategy (Shimura et al. 1999). In the case of Hs soil sample, five rRNA morphotypes were identified. These bacteria were grouped into the genera *Burkholderia*, *Myroides* and *Bacillus*. The majority of isolates corresponded to the genus *Burkholderia*. Member of this genus are generally known for their ability to produce exopolysaccharide, which aid to alleviate the negative effect of highly toxic pollutants. *Burkholderia* have clusters of *bph* genes in their genome that give it the biochemical capacity to degrade biphenyl/PCB (Witzig et al. 2006). In the soil sample Hp, there were three different bacterial species that corresponded to the genera *Burkholderia* and *Bacillus.* The strains DCB07 had 97.0% of genetic to *Burkholderia anthina* MYSP113, while strains DCB08 showed 95.0% similarity to *B. vietnamiensis* BU97 and DCB12 had 98.3% similarity to *Bacillus anthracis*. The ability to degrade PCBs by *Burkholderia*, it has already been documented (Bartels et al. 1999, Denef et al. 2004; Agulló et al. 2007). It oxidizes more than 20 PCB congeners including some with 4, 5 and 6 chlorine substitutions on the biphenyl rings. This bacterium is characterized by having *bph* genes that encode enzymes that participate in the biphenyl degradation pathway. Also, *Bacillus* have been reported as PCB-degrading bacteria. For instance, thermophilic *Bacillus* sp. JF8 showed degradation of PCB congeners including tetra- and penta-chlorobiphenyl and naphthalene (Hatta et al. 2003) and the *Bacillus cereus* JP12 that had the capacity to degrade decabromodiphenyl ether (Lu et al. 2013).

Regarding the soil samples F and A, which were contaminated *in vitro* by mixtures of aroclor, different bacterial species were identified. In sample F, five different ARDRA morphotypes were identified and grouped into the genera *Acinetobacter*, *Kosakonia*, *Comamonas*, *Enterobacter* and *Staphylococcus*. All of these bacteria have been recognized for their ability to PCBs and other highly chlorinated chemical compounds. As the case of *Comamonas testosteroni* who had the ability to degrade more than 95% of PCBs. Also, Qiu et al. (2015) showed that *C. testosteroni* can degrade decachlorobiphenyl (PCB209) in cold conditions. In sample A, the isolates were affiliated to the *Acinetobacter*, *Burkholderia*, *Comamonas* and *Enterobacter* genera. These bacteria have genetic characteristics for potential degradation of PCBs. Liang et al. (2014) identified high abundance of *Acinetobacter* in sediments contaminated by PCBs where there was a dechlorination of these contaminants. Also, *Enterobacter* sp. LY402 isolated from polluted soil efficiently degrade PCBs under aerobic conditions, where biphenyl dioxygenase is the key enzyme in the PCBs biodegradation process (Cao et al. 2011).

PCA analysis based on the relative abundance of the different bacterial phylotypes (Fig. 3) corroborated the effect of the PCB contaminant in the different samples studied. Samples Hs and Hp had a marked separation with samples F and A, possibly due to the effect of concentration and exposure time of PCB. In the soils Hp and Hs anthropogenically contaminated by high concentrations of PCBs, significant variations were observed in some parameters related to fertility. Total C, total N, and total P and mainly SOM influenced in the functionality of the biogeochemical cycles that in turn favored the increase in the relative abundance of oligotrophic bacteria, grouped in the Phyla *Firmicutes*, *Verrucomicrobia* and *Plantomycetes*. While samples F and A were related to copiotrophic species, grouped into the phyla *Actinobacteria* and *Proteobacteria.* The high concentrations of the aroclor mixtures influenced the bacterial communities by exerting selective pressure, in such a way that only those bacterial species with specialized metabolism survive in these extreme conditions. This selection process is usually variable with respect to the PCB exposure time in the affected soils. In this condition, bacteria have the ability to use biphenyls as a source of C and carry out degradation and mineralization processes through a variety of biochemical pathways, thereby establishing new abiotic conditions that influence diversity and structure of bacterial communities in the ecosystem (Mulligan and Yong 2004). Mikolasch et al. (2019) studied the effect of the diversity of microorganisms in soils contaminated by PCBs, finding dominant bacteria that metabolize macromolecules, making them assimilable compounds for other bacteria that influence the degradation of recalcitrant contaminants. However, it is important to consider that in contaminated soils, fungi and other microorganisms play an important role in the degradation of toxic chemical compounds.

Principal component analysis allowed establishing a correlation between the characteristics of soils contaminated by PCBs with respect to the specific abundances of the different phylotypes identified (Fig. 4). The anthropogenic samples were grouped (PC1 became mostly negative) and is related to the concentration of aroclor 1260 containing the most recalcitrant congeners of PCBs (Wahlang et al. 2014) and the C:N ratio that is influenced by the breakdown of microbial waste and cellular respiration that indicates the presence of bacterial metabolism despite the presence of the contaminant (Wu et al. 2017), while samples F and A had a mostly positive PC1 with the rest of the soil parameters (total N, total C, total P, pH and organic matter) relating it to the aroclor mixtures 1242 and 1254 that correspond to the easiest assimilation congeners for bacterial metabolism (Martinez 2010). This indicates the importance of different nutrients for bacterial metabolic activities focused on the assimilation of pollutants and the effect on relative abundances (in the case of soils F and A) and as the nutrients are depleted by bacteria with oligotrophic characteristics and with the potential to assimilate and metabolize PCBs, they play an important and crucial role in natural attenuation processes for the remediation of soils contaminated by PCBs.

In this study, a wide diversity and abundance of bacterial species grouped in the phyla *Proteobacteria*, *Firmicutes* and *Flavobacteriia* were identified in the bacterial communities that inhabit soils contaminated by high concentrations of PCBs. *Acinetobacter*, *Bacillus*, *Burkholderia* and *Comamonas* were the genera with the highest abundance, both in samples collected in anthropogenically contaminated Hs and Hp soils, as well as in those samples contaminated *in vitro* by PCBs, this according to the indices of abundance and diversity using both cultivable and non-cultivable approaches. The relative abundance of some species varied significantly as the PCB content in the soils increased. The bacterial communities correlated positively with some chemical parameters (mainly with organic matter) and there was also a relationship with pH, total C, total N and total P, which influences bacterial metabolic processes and also the mineralization of organic matter and possibly of the PCBs (as C source). Several of the isolated strains had genetic similarity with bacterial species, such as *Bacillus cereus*, *Comamonas testosteroni* and *Burkholderia cepacia* who possess a cluster of *bph* genes involved in PCB/biphenyl degradation and for this reason, these bacteria become important biotechnologies for use in programs aimed at the bioremediation of soils contaminated by PBC through biostimulation and bioaugmentation processes.

## Supplementary information

**Additional file 1: Table S1.** Geographical location of sampling sites.

## Data Availability

We admit availability of data and material.
