# Supplementary material for: Structure and diversity of native bacterial communities in soils contaminated with polychlorinated biphenyls
Source: AMB Express. 2020 Jul 10;10:124. doi: 10.1186/s13568-020-01058-8 (PMC7351888; doi:10.1186/s13568-020-01058-8)
Supplement: Supplementary file 1 — Additional file 1: Table S1. Geographical location of sampling sites. [file 13568_2020_1058_MOESM1_ESM.docx]

**Table S1** Geographical location of sampling sites

| ______________________________________________________________________________________________ | | | | |
| --- | --- | --- | --- | --- |
| Sample | Sample source | Site name | Geographic location  (Altitude) | Collection date |
| ________________________________________________________________________________________________ | | | | |
| Control | Without PCB | “La Herradura”  (Malpaso, Chiapas, Mexico) | 17.11 °N, 93.36 °W  (136 masl) | 05-February-2019 |
| Hs | PCB contaminated  soil | “La Herradura”  (Malpaso, Chiapas, Mexico) | 17.11 °N, 93.36 °W  (136 masl) | 05-February-2019 |
| Hp | PCB contaminated  soil | “La Herradura”  (Malpaso, Chiapas, Mexico) | 17.11 °N, 93.36 °W  (136 masl) | 05-February-2019 |
| F | Forestal  soil | “El Ocote”  (Ocozocoautla, Chiapas, Mexico) | 16.81°N, 93.37 °W  (985 masl) | 21-February-2019 |
| A | Agricultural  soil | “La Gloria”  (Suchiapa, Chiapas, Mexico) | 16.11 °N, 93.06 °W  (620 masl) | 11-February-2019 |
| ___________________________________________________________________________________________ | | | | |
